# Bacteria colonising *Penstemon digitalis* show volatile and tissue-specific responses to a natural concentration range of the floral volatile linalool

**DOI:** 10.1007/s00049-018-0252-x

**Published:** 2018-03-01

**Authors:** Rosalie C. F. Burdon, Robert R. Junker, Douglas G. Scofield, Amy L. Parachnowitsch

**Affiliations:** 1 Department of Plant Ecology and Evolution, Evolutionary Biology Centre, Uppsala University, Norbyvägen 18d 75236 Uppsala, Sweden; 2 Department of Biosciences, University Salzburg, Hellbrunnerstr. 34 5020 Salzburg, Austria; 3 Department of Evolutionary Biology, Evolutionary Biology Centre, Uppsala University, Norbyvägen 18d 75236 Uppsala, Sweden; 4 Uppsala Multidisciplinary Center for Advanced Computational Science, Uppsala University, 75105 Uppsala, Sweden

**Keywords:** Anti-microbial, Phyllosphere, Plant defence, Scented nectar, Volatile organic compounds (VOCs)

## Abstract

Bacteria on floral tissue can have negative effects by consuming resources and affecting nectar quality, which subsequently could reduce pollinator visitation and plant fitness. Plants however can employ chemical defences to reduce bacteria density. In North American, bee-pollinated *Penstemon digitalis*, the nectar volatile *S*-(+)-linalool can influence plant fitness, and terpenes such as linalool are known for their antimicrobial properties suggesting that it may also play a role in plant–microbe interactions. Therefore, we hypothesized linalool could affect bacterial growth on *P. digitalis* plants/flowers. Because *P. digitalis* emits linalool from nectar and nectary tissue but not petals, we hypothesised that the effects of linalool could depend on tissue of origin due to varying exposure. We isolated bacteria from nectary tissue, petals and leaves, and compared their growth relative to control using two volatile concentrations representing the natural emission range of linalool. To assess whether effects were specific to linalool, we compared results with the co-occurring nectar volatile, methyl nicotinate. We show that response to floral volatiles can be substance and tissue-origin specific. Because linalool could slow growth rate of bacteria across the *P. digitalis* phyllosphere, floral emission of linalool could play a role in mediating plant–bacteria interactions in this system.

**Electronic supplementary material:**

The online version of this article (10.1007/s00049-018-0252-x) contains supplementary material, which is available to authorized users.

## Introduction

Microbes colonising plant tissues are common and can have a range of effects from mutualistic to antagonistic. In the context of flowers and nectar, these microbes can also influence interactions with mutualistic pollinators (Aleklett et al. 2014). While nectar yeasts may attract pollinators (Schaeffer and Irwin 2014), thus far nectar bacteria have shown negative effects on pollinator-interactions (Vannette et al. 2013; Good et al. 2014). However, plants are not helpless and can deploy defences against microbes colonising important reproductive structures, such as emitting floral volatiles with anti-microbal properties (Junker and Tholl 2013). For example, in *Arabidopsis thaliana* the floral volatile (*E*)-*β*-caryophyllene can directly inhibit the growth of pathogenic bacteria colonising stigma tissue (Huang et al. 2012). Common floral volatiles such as terpenoids and nitrogen-containing alkaloids also have antimicrobial properties (Griffin et al. 1999; Sansores-Peraza et al. 2000; Queiroga et al. 2007) but these are often tested against lab bacteria strains rather than ecologically relevant bacteria. More generally, antimicrobial activity of essential oils rich in volatiles common to both vegetative and floral tissues is well known (Hammer et al. 1999; Dorman and Deans 2000; Höferl et al. 2009) and volatiles can also mediate microbe interactions in soil (Effmert et al. 2012), suggesting that volatiles in floral scent bouquets may play a role in plant–microbe interactions.

Flowers generally emit the highest amounts and most diverse blends of volatile organic compounds (VOCs) across plant organs (Knudsen et al. 2006; Dudareva et al. 2013). Differences in chemical composition across the phyllosphere could enforce selective environments for the establishment and growth of microorganisms (Del Giudice et al. 2008; Junker et al. 2011). For example, the floral volatiles phenylacetonitrile and 2-phenylethyl alcohol inhibit growth of bacteria originating from *Saponaria officinalis* leaves much more efficiently than bacteria isolated from flowers of the same species, suggesting that floral volatiles could influence bacteria composition among tissues (Junker et al. 2011). It may be particularly important to control bacteria found in nectar rewards because bacteria can degrade nectar or effect flower signals such as scent and subsequently affect pollination (Canto and Herrera 2012; Vannette et al. 2013; Good et al. 2014; Rering et al. 2017; Helletsgruber et al. 2017). Therefore, emission from specific tissues such as flowers or volatiles in nectar could affect in the reproductive success of plants via microbe interactions (Aleklett et al. 2014).

Although volatiles can have anti-microbial properties, microbe responses to secondary metabolites can be strain- and volatile-specific, or dependent on volatile concentration (Junker and Tholl 2013; Vannette and Fukami 2016). For instance, caffeine inhibits the growth and density of nectar microbes specifically at high concentrations, whereas aucubin increases the growth rate of microbes (Vannette and Fukami 2016). Furthermore, not all plant–microbe interactions are anti-microbial in nature; some bacteria found in the soil are adapted to metabolise VOCs as a carbon source (Kleinheinz et al. 1999; Del Giudice et al. 2008), but this is yet to be shown for bacteria in the phyllosphere. Therefore, we tested whether floral volatiles can suppress or facilitate the growth and density of plant-associated bacteria. Additionally we tested whether bacteria could use floral volatiles as a carbon source for growth.

We isolated bacterial communities from the leaves, petals, and nectary tissue of *Penstemon digitalis*, which vary in their exposure to (*S*)-(+)-linalool (hereafter ‘linalool’) emitted from the nectary and nectar (Parachnowitsch et al. 2013; Burdon et al. 2015). We hypothesized that field-collected bacteria colonizing *P. digitalis* plant tissues could differ in their response to the presence of linalool because they are exposed to different levels in natural conditions. From each tissue, we randomly selected individual bacterial strains to assess their growth rate and maximum density in control and floral volatile-exposed conditions. We focused our investigations on linalool for four reasons: first, linalool is one of the most common volatiles emitted as floral scent across angiosperms (Knudsen et al. 2006); second, linalool has been shown to have antimicrobial properties (Queiroga et al. 2007): third, in *P. digitalis* linalool was previously identified as a target of phenotypic selection with high emitters having increased reproductive success (Parachnowitsch et al. 2012); and fourth, of the abundant scents making up the floral bouquet only linalool can directly protect nectar because it is also found in the nectar (Parachnowitsch et al. 2013). We tested linalool’s antimicrobial or facilitation effects at a concentration range based on field emissions. To determine whether any effects were due to linalool or simply due to the presence of any floral volatile, we compared linalool’s effects to that of methyl nicotinate, using the same concentration range. While methyl nicotinate effects on plant reproduction and emission strength are unknown for *P. digitalis*, it is an ecologically relevant comparison because methyl nicotinate has been detected in targeted scent collections from nectar (Burdon et al. 2015). However, because methyl nicotinate is not detected in general floral bouquet sampling (Parachnowitsch et al. 2012), it suggests the concentration is much lower than linalool’s within the flower.

## Methods and materials

### Bacteria isolation and identification

We sampled three distinct bacterial microhabitats from the flowers and leaves of *Penstemon digitalis* plants (*n* = 3 plants: 2 flowers (one male phase and one female phase to cover the age span of the flowers) and 1 leaf per plant, Ithaca, New York, Aug 2014). For each flower, flower corollas were separated into two parts, the scentless flower petals and the volatile-emitting nectary (Burdon et al. 2015). Because *P. digitalis* produces relatively low amounts of nectar (Junker and Parachnowitsch 2015), samples from the nectar alone were difficult to obtain from the field. A single sample yielded one bacteria strain that was identified (see “Results”), but was not included in further tests due to lack of replication. However, when collecting nectar, pollinators encounter bacteria in the corolla tube along with nectar due to the constricted corolla tube of *P. digitalis* so whole-nectary samples represent what bacteria could be dispersed into/found in the nectar. Tissue samples were preserved individually in 1.5 ml Screw Cap micro tubes (BRAND^®^, Sigma Aldrich) containing 500 µl lysogeny broth (LB). All sampling equipment was sterilized with ethanol before sampling. Bacteria were allowed to grow for 12 h prior to adding 750 µl glycerin and preserving at − 80 °C until use. To separate bacteria from plant material, tissues were sonicated (7 min) and then vortexed. A 2 µl 1:100 LB dilution of each sample was grown on LB agar plates (LB-Medium Powder, AppliChem, Darmstadt, Germany; Bacto Agar, Becton, Dickinson and Company, Sparks, USA), containing fungicide Cycloheximide (Sigma–Aldrich, Steinheim, Germany, 30 µg/l) (Junker et al. 2014). After plate incubation at 24 °C for 72 h, bacterial strains were distinguished based on colour, shape, reflectance and texture. Distinct morphs (hereafter ‘strains’) were cultivated on separate LB agar plates containing no fungicide (*n* = 81), of which 47 strains were randomly selected for identification.

To identify bacteria, DNA was extracted and multiplied by PCR using primers to anneal with conserved regions of bacterial 16S rRNA genes. The lysates were centrifuged at 14,000*g* for 3 min and the supernatant containing bacterial DNA acted as template for polymerase chain reaction (PCR). DNA of one colony per strain was extracted using the High Pure PCR Template Preparation kit (Roche, Grenzach-Wyhlen, Germany) following the manufacturer’s instructions. The following conditions were used for extraction: 1 µl genomic DNA was dissolved in 21.9 µl sterile distilled water (dH20), 6 µl of 10-fold reaction buffer (Moltaq PCR kit, Molzym GmbH and Co.KG, Bremen, Germany) and 10 mM dNTP-mix (Thermo Scientific, Munich, Germany). We added 1 µl of forward primer (27f), 1 µl of reverse primer (1492r) (Metabion, Martinsried, Germany) and 0.3 µl Taq polymerase (Molzym) giving a total volume of ~ 30 µl per sample (Junker et al. 2014). A thermocycler (Eppendorf Mastercycler gradient, Hamburg, Germany) with the following programme was used: initial denaturation at 94 °C for 3 min, 35 cycles of 94 °C for 30 s, 52 °C for 30 s, 72 °C for 100 s and a final extension step at 72 °C for 5 min. Positive and negative controls with and without genomic DNA were made for each set of samples during the PCR. PCR products were purified with the Wizard SV Gel and PCR clean-up kit (Promega, USA) according to the instructions of the producer. DNA concentration was measured using a NanoDrop-ND-1000 (NanoDrop Technologies, Wilmington, USA), and a total of 375 ng of DNA along with 2 µl primer 1492r (20 pM) were sent to Eurofins Genomics (Ebersberg, Germany) for Extended HotShot sequencing. Sequences were quality start- and end-trimmed according to phred scores with 4Peaks (Nucleobytes, Amsterdam, NL) (see Junker et al. 2011 for further experimental detail). Bacterial strains (*n* = 47) were taxonomically assigned to the lowest taxonomical level possible via the GenBank nucleotide database (accessed 20th February 2015) (Benson et al. 2013): Accession numbers: KX891497–KX891543.

### Volatile bioassays and volatiles as a carbon source

To determine the effect of linalool and methyl nicotinate on bacterial growth rate and maximum density, we tested bacteria strains using natural linalool emissions of low (5 ng/ml) and high (100 ng/ml) concentrations in basic nutrient media (SRM + glucose) (Del Giudice et al. 2008). Media consisted of 0.75 g ammonium dihydrogen phosphate (Merck, Darmstadt, Germany), 0.15 g potassium chloride salt (Roth, Karlsruhe, Germany), and 0.15 g magnesium sulphate (Alfa Aesar GmbH, Karlsruhe, Germany) for SRM and 0.75 g glucose (Sigma Aldrich^®^)/750 ml distilled water (unless otherwise noted). Prior to resolved identification of the strains we randomly selected and tested eight nectary, ten petal and five leaf strains (*n* = 23). Constraints for growing bacteria prevented testing all 47 strains and one selected strains failed to grow (see Table 1, giving final *n* = 22). To determine volatile concentrations that reflect plant emissions, we used measurements of linalool emissions from two common gardens of *P. digitalis* in 2012 from the same populations where we collected bacteria samples and other base-line data on the species (Parachnowitsch et al. 2012; Burdon et al. 2015). The average linalool emitted from 86 *P. digitalis* inflorescences was 44 ± 4 ng with a range from < 5 ng to few plants producing over 100 ng. We chose to use the same concentration range for methyl nicotinate as a direct volatile treatment comparison, although it is emitted at much lower amount than linalool in *P. digitalis* flowers (Burdon et al. 2015). We conducted two types of volatile bioassay: (1) to test for suppression/facilitation of volatiles, bioassays contained either low or high volatile concentrations (5 vs 100 ng) in SRM + glucose media and (2) to test whether bacteria could metabolise volatiles as a carbon source bioassays were run with volatiles only and no glucose. As a control, we also tested all strains SRM + glucose media without volatiles. Racemic linalool and methyl nicotinate used were from Sigma Aldrich^®^.

**Table 1:** Bacterial strain identification

Species	Number of strains (number tested)	BLAST identity	References
Leaf tissue			
*Bacillus safensis*	5 (3)	1	Identified from spacecraft surfaces (Satomi 2006) and detected in *Asphodelus aestivus* and *Capsodes infuscatus* nectar (Samuni-Blank et al. 2014) as well as *Citrus paradisi* (Fridman et al. 2012)
*Pantoea agglomerans*	1 (1)	0.99	Thought to be non-pathogenic to plants and used as biocontrol agent (Johnson et al. 2000) and found in *Amygdalus communis* (Fridman et al. 2012) and *Capsodes infuscatus* nectar (Samuni-Blank et al. 2014)
*Pantoea eucalypti*	1 (1)	0.99	Isolated from blight in eucalyptus (Brady et al. 2009) and found in nectar (Samuni-Blank et al. 2014)
*Pseudomonas oryzihabitans*	1 (0)	0.98	Growth promotion for potato plants by iron capture (Sessitsch et al. 2004)
Petal tissue			
*Erwinia aphidicola*	2 (0)	0.99	Pathogenic to aphids and plants (Grenier et al. 2006; Santos et al. 2009)
*Erwinia persicina*	1 (0)	0.99	Non-pathogenic (Hao et al. 1990) and found in nectar (Fridman et al. 2012; Samuni-Blank et al. 2014)
*Erwinia* sp.	1 (0)		
*Ewingella americana*	2 (2)	0.97–0.99	Pathogenic to mushrooms (Inglis et al. 1996)
*Pantoea agglomerans*	7 (2)	0.98–0.99	See above
*Pantoea eucalypti*	4 (2)	0.97	See above
*Pantoea vagans*	2 (1)	0.99	Biocontrol against fire blight (Kamber et al. 2012)
*Rosenbergiella collisarenosi*	1 (growth failed)	0.99	Isolated from floral nectar (Lenaerts et al. 2014)
Nectary tissue			
*Acinetobacter bereziniae*	1 (1)	0.80	Non-pathogenic, evidence for plant growth promotion (Martínez-Rodríguez et al. 2014)
*Acinetobacter nectaris*	3 (1)	0.98	Isolated from nectar (Álvarez-Pérez et al. 2013; Jacquemyn et al. 2013; Samuni-Blank et al. 2014; Bartlewicz et al. 2016)
*Erwinia rhapontici*	1 (1)	0.93	Pathogenic to many plants (Feistner et al. 1983)
*Pantoea agglomerans*	5 (5)	0.98–0.99	See above
*Pantoea ananatis*	1 (0)	0.99	Common plant pathogen in agricultural crops and forest tree species worldwide (Coutinho and Venter 2009)
*Pantoea eucalypti*	1 (0)	0.99	See above

To prepare the bacteria samples, we transferred one colony per bacterial strain from agar plates into 1 ml volatile treated/control SRM and took an initial optical density measurement at 600 nm (OD_600_) (Biotek ELX808, software Gen5 version 2.04). Bacteria solutions were transferred into 96-well microwell plates (A. Hartenstein) (*n* = 7 replicates/strain, 11–12 strains/plate), with a standardised initial OD_600_ of 0.01 across strains and treatments. Per 96-well microwell plate, we included a media only row as a within-plate control. To prevent bacterial settling, plates were kept incubated at 28 °C and gently shaken at 270 rpm (Grant-bio PHMP-4 Thermo-Shaker). Optical density measurements were taken hourly (0–12 h) to determine growth rate and recorded 3–6 h thereafter until bacteria reached an asymptote (within 30 h). The lids of the plates were treated with Triton X-PO in 20% ethanol (Sigma Aldrich^®^) to prevent condensation and false OD readings. The above methods were repeated with SRM without glucose to assess if any bacterial strain could use linalool or methyl nicotinate as an alternative carbon source. The basic media was autoclaved (125 °C/35 min) prior to volatile inoculation.

### Statistical analysis

We fit bacterial growth curves using the R package grofit (Kahm et al. 2010) as has been used in other studies for nectar microbes (e.g. Vannette and Fukami 2016). Grofit selects the best fit model using Akaike’s Information Criterion (AIC) and these were visually confirmed, and where appropriate, model returns of NA were adjusted to zeros to reflect where bacteria did not grow. We used the estimated parameters µ (h^−1^) the maximum growth rate and *A* [OD_600_] the asymptote of the growth curve, which is the maximum density reached. Both growth rate and maximum density likely influence the ability of bacteria to compete and disperse in ephemeral floral tissues/nectar.

To assess the response of bacteria to linalool and methyl nicotinate relative to the control, we fit mixed-effect models for growth rate (*µ*) or density (*A*) as the response variable and volatile concentration (control, 5 ng, 100 ng) × tissue origin (leaf, petal, nectary) as explanatory variables and bacteria strain, genera, and species as random effects. To assess differences among treatments within tissue origin, we ran separate ANOVAs for each tissue type followed by Tukey post hoc tests. We excluded from analysis *Rosenbergiella collisarenosi* because fewer than three replicates’ growth curves could be estimated in the control. We performed separate mixed effect models for linalool and methyl nicotinate, however, to test whether these two volatiles differed in their effects we grew the strains with 100 ng of either volatile at the same time and compared these. No strain of bacteria grew in the volatile without glucose treatments and so no further analysis was conducted using these data. Statistical analyses were performed in *R* (R Core Team 2017).

## Results

### Bacteria isolation and identification

From the phyllosphere of *P. digitalis* plants we identified 13 species of bacteria representing 7 genera (Table 1). From leaf surfaces we identified four species of bacteria from eight isolated strains, from petals seven species from 20 isolated strains and from nectary tissue seven species from 19 strains. The most ubiquitous species were *Pantoea agglomerans* and *P. eucalypti*, which were present on both leaves and flowers. Leaf tissue strains also included *Bacillus safensis* and *Pseudomonas oeyzihabitans* that were lacking on the flower samples. In addition to *P. agglomerans* and *P. eucalypti*, both petal and nectary tissue had members of the genus *Erwinia* but with different species. The scentless petals also had species of *Ewingella*, and *Rosenbergiella* while species of *Acinetobacter* were only detected in the nectary samples and the one strain from nectar was also *A. nectaris*. Although we found genera composition differed between tissues (Table 1), the differences in bacterial colonization between the tissues are based on few cultivatable strains; further assessments of bacterial communities from cultivation-independent methods would be required to examine differences in tissue diversity.

### Volatile bioassays

**Fig. 1:**
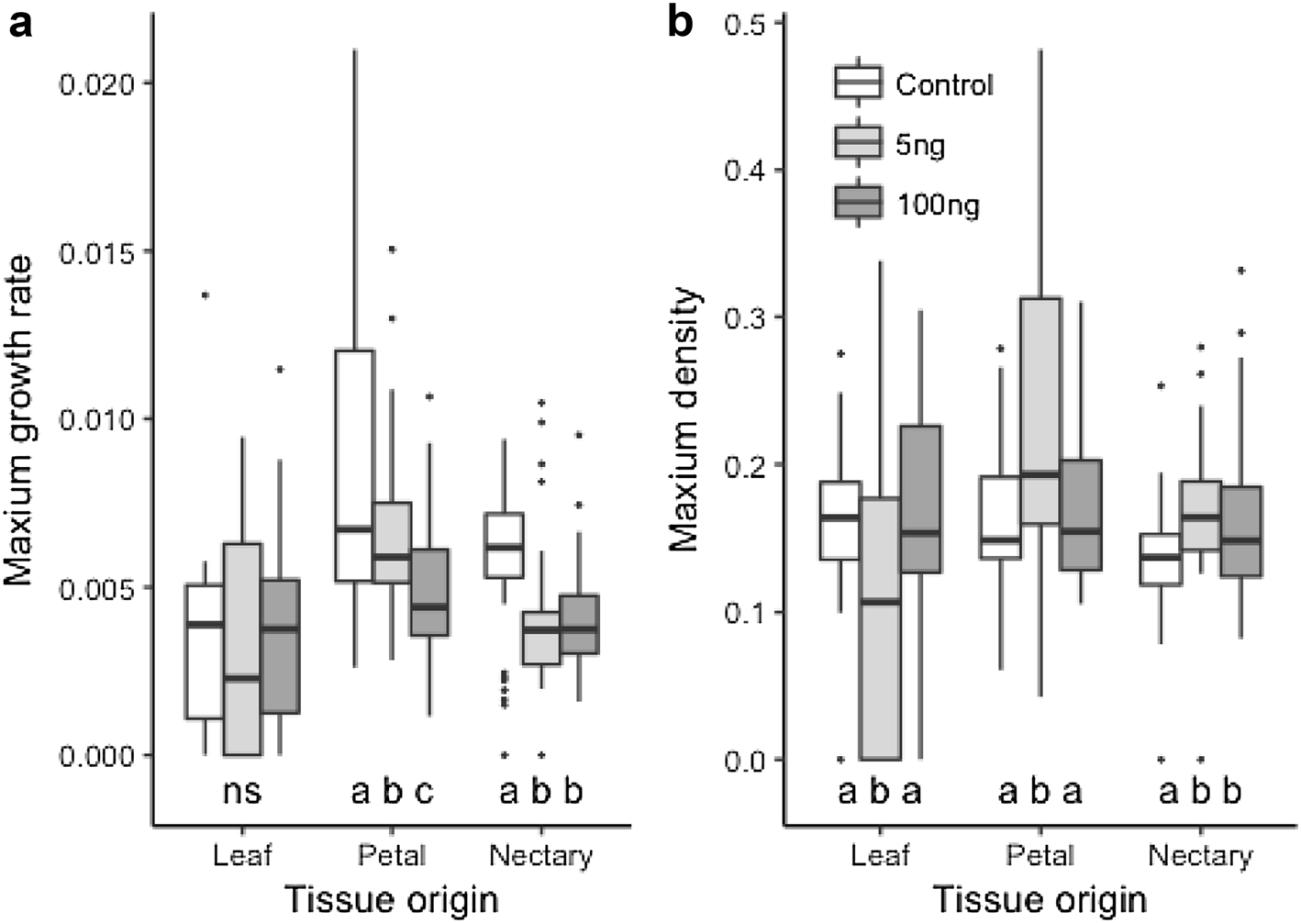
**a** The maximum growth rate (*µ*) and **b** maximum density (*A*) for bacteria strains isolated from *P. digitalis* plants cultured in control or with the floral volatile linalool. Linalool concentrations are ecologically relevant for inflorescence emission variation and tissue origin arranged from low emitting tissues (leaf) to high (nectary). Statistical tests in Table 2; we show the boxplots to demonstrate the full range of the data, means, standard errors, and sample sizes found in Supplementary Table 1. Letters represent within tissue post hoc tests to determine differences among growth media

In general, linalool had a greater effect on bacteria than methyl nicotinate and the effects were tissue-origin specific. While bacteria isolated from leaves were not affected by linalool, growth was slower for both petal and nectary isolates when linalool was added (Fig. 1; Table 2). Likewise, the maximum density of leaf isolates was not affected by linalool, while for petal and nectary isolates the low linalool treatment actually increased maximum density (Fig. 1; Table 2). Linalool concentration did not have an additive effect on either growth or density; slower growth at higher linalool concentration was only seen for petal isolates and only small amounts of linalool facilitated increase density. Overall leaf isolates did more poorly than those from floral tissues.

**Table 2:** ANOVA table of fixed effects for bacterial growth and density in control, 5 and 100 ng volatile conditions

Model	Fixed effects	*F*	*P*
Linalool models (*N* = 349)			
Maximum growth	Volatile concentration	*F*_2,348_ = 33.03	< 0.001
	Tissue origin	*F*_2,348_ = 6.28	0.011
	Volatile × tissue	*F*_4,348_ = 4.17	0.0026
Maximum density	Volatile concentration	*F*_2,348_ = 4.39	0.013
	Tissue origin	*F*_2,348_ = 5.38	0.023
	Volatile × tissue	*F*_4,348_ = 10.36	< 0.001
Methyl nicotinate models (*N* = 377)			
Maximum growth	Volatile concentration	*F*_2,376_ = 9.83	< 0.001
	Tissue origin	*F*_2,376_ = 5.41	0.018
	Volatile × tissue	*F*_4,376_ = 8.86	< 0.001
Maximum density	Volatile concentration	*F*_2,376_ = 2.91	0.056
	Tissue origin	*F*_2,376_ = 5.72	0.015
	Volatile × tissue	*F*_4,376_ = 1.50	0.20

Strain, genus and species were included as random effects

**Fig. 2:**
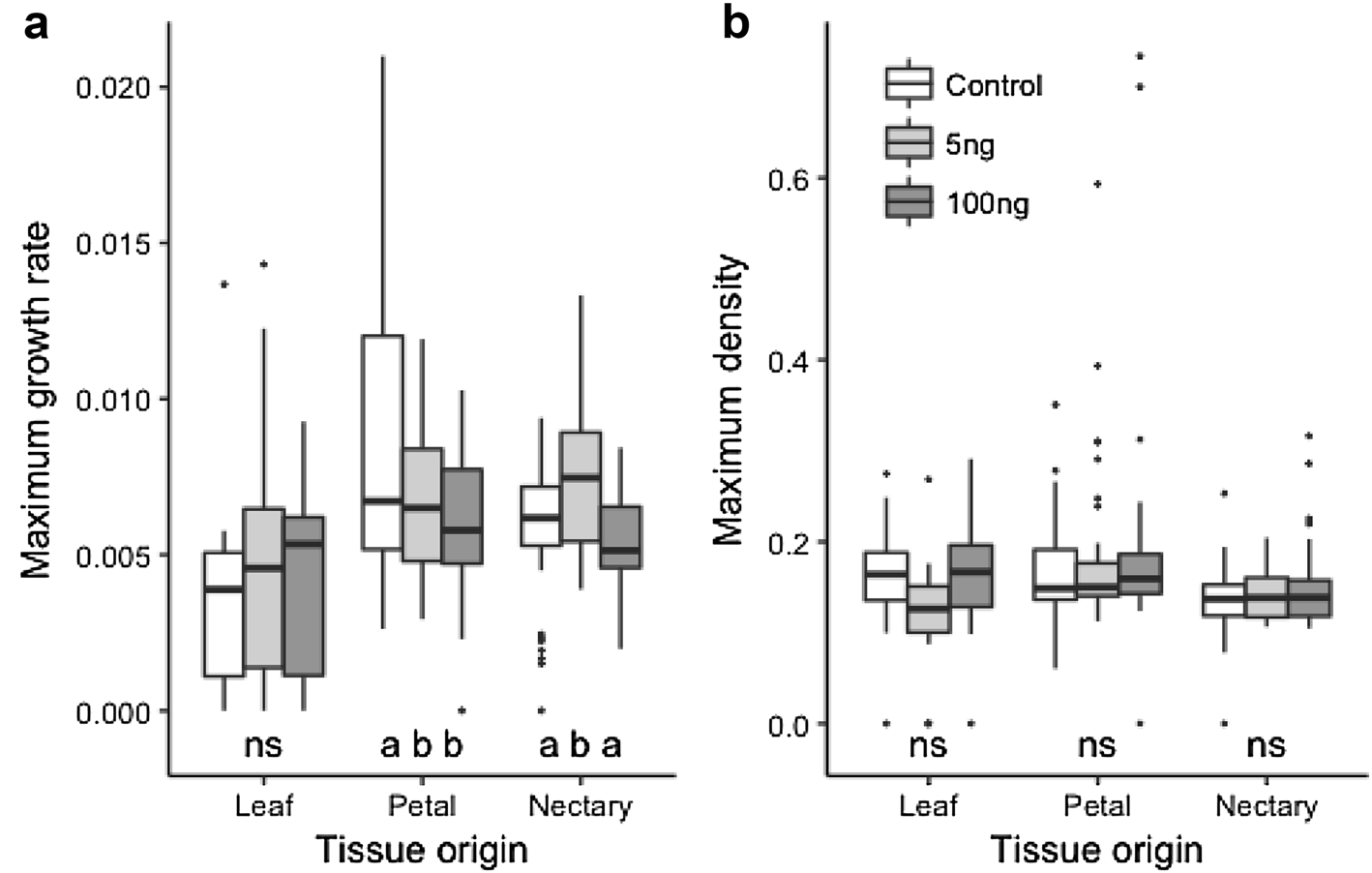
**a** The maximum growth rate (*µ*) and **b** maximum density (*A*) for bacteria strains isolated from *P. digitalis* plants cultured in control or with the nectar volatile methyl nicotinate. Concentrations reflect the range for linalool emission for comparison, and are much higher than natural nectar emission of methyl nicotinate in *P. digitalis*; control treatment is the same as Fig. 1. Statistical tests in Table 2; means, standard errors, and sample sizes found in Supplementary Table 1. Letters represent within tissue post hoc tests to determine differences among growth media

Methyl nicotinate had little effect on bacterial growth rate or maximum density. As was seen for linalool, leaf isolates were never strongly affected by methyl nicotinate but there was some reduction in growth rate at the ecologically high levels of methyl nicotinate for petal and nectary isolates (Fig. 2; Table 2). Unlike linalool, maximum density was not affected by methyl nicotinate (Table 2). When growing bacteria in high concentrations of linalool and methyl nicotinate, we found a significant difference in maximum growth between the substances (*F*_1,236_ = 31.79, *P* < 0.001) but not density (*F*_1,236_ = 0.079, *P* = 0.78).

## Discussion

In our study, leaves and flowers of *P. digitalis* were colonized by bacteria commonly described in other plants (Table 1), although plant species can differ substantially in the bacteria detected, for example in nectar (Álvarez-Pérez et al. 2012). Addition of linalool inhibited bacterial growth rate significantly compared to control media and methyl nicotinate treatments. Antimicrobial effects of linalool have been shown previously (e.g. Queiroga et al. 2007; Kamatou and Viljoen 2008; Taniguchi et al. 2014; Herman et al. 2016) and our results show that linalool can also affect bacteria collected from wild plants at ecologically relevant concentrations. In particular, as a nectar component, linalool could play a more complex role in plant–pollinator interactions if its effects on nectar microbes alter pollinator behaviour. However, linalool might even facilitate density for some bacteria at low concentrations (Fig. 1) suggesting more detailed experiments are needed to tease apart its role. Furthermore, our study focused on bacteria, while yeasts are also commonly to nectar and linalool may affect their growth in this system. More generally, *Penstemon* whole plant extracts show antimicrobial activity (Zajdel et al. 2012a, b, 2013) and our results suggest that floral volatiles, at least linalool, may be an additional important compound for plant–microbe interactions.

It is often assumed the primary function of floral volatiles is the attraction of pollinators (Wright and Schiestl 2009). However, floral volatiles are increasingly being shown to aid floral defence (Kessler and Baldwin 2007; Theis et al. 2007; Junker and Bluthgen 2008), including defence against surface-dwelling pathogens (Huang et al. 2012). Bacteria are known to degrade nectar sugars, alter nectar pH (Herrera et al. 2008; Vannette et al. 2013) and nectar microbes can disrupt pollination and reduce plant fitness (Junker et al. 2014). Defending floral tissues from such microorganisms could thus be crucial to plants (Fridman et al. 2012; Huang et al. 2012; Bulgarelli et al. 2013). Our work supports the hypothesis that in addition to attracting pollinators or repelling antagonists, floral scents could play a role in plant–microbe interactions. For example, *P. digitalis* increases emission strength of linalool during the day possibly to attract day active pollinators such as bumblebees (Burdon et al. 2015), however daytime is also when temperatures rise and conditions facilitate floral bacterial growth.

We found the effects of floral volatiles on bacterial growth rate or maximum density often depended on tissue origin. Strain specific variation in growth rate to different volatiles or concentrations is not new (Vannette and Fukami 2016). For bacteria, different genera and strains/species within genera can differ in metabolic capability, nutritional requirements and adaption to environmental stresses including oxidative stress from VOCs (Lindow and Brandl 2003; Lievens et al. 2015). For example, *A. nectaris* appears to be a nectar specialist that metabolises specific sugars and amino acids at temperatures between 25 and 30 °C compared with other *Acinetobacter* species (Álvarez-Pérez et al. 2013). Here, we found floral bacteria strains were generally more sensitive to floral volatiles compared to leaf strains. Thus, tolerance to floral volatiles could impact bacterial strain establishment on different tissues and affect bacterial community composition or competitive ability within communities (Lindow and Brandl 2003; Lievens et al. 2015). Our results ran contrary to our hypothesis, suggesting that those bacteria found in close proximity to linalool production are not tolerant and its presence may modulate bacterial growth in the flower and nectar by slowing growth. However, maximum density was not suppressed by linalool (or methyl nicotinate) suggesting that volatile effects on microbial communities need further investigation to understand their impacts within the flower, especially in a constantly changing resource such as nectar. One possibility is bacterial growth rate may be more important for determining successful colonization of wildflowers than maximum density because nectar resources are ephemeral and microbe communities may not have time to reach maximum density. However, we need relevant field testing to assess whether these factors help explain differences in effects of linalool in our system.

We found none of the isolated bacterial strains metabolized volatiles as their sole carbon source (not shown because all had zero growth), but some strains grew more with volatiles present when concentrations were low when sugar was also available. If floral volatiles are employed as a microbial defence, bacteria tolerance to volatiles could have detrimental effects on plant fitness. For example, nectar specialist microbes can alter floral volatiles and subsequent plant–pollinator interactions (Vannette and Fukami 2016; Rering et al. 2017; Helletsgruber et al. 2017). While we did not measure volatile concentration after bacterial growth, the nectar specialist *A. nectaris* is the most likely candidate to affect volatile strength in nectar. In this scenario an arms race between plants and microbes could drive selection on increased strength of volatiles emitted by the plant similar to the selection on linalool we observed in the field (Parachnowitsch et al. 2012). Future work should explore if bacteria are capable of driving selection on VOCs by comparing how suppression or facilitation of bacteria can negatively or positively affect plant reproduction (Huang et al. 2012; Junker and Tholl 2013; McArt et al. 2014).

Our lab experiments may not represent tissue communities on *P. digitalis* plants for four reasons. First, cultivation technique likely biased the bacteria included in our study because all isolation media, extraction procedures and incubation methods are selective to some extent. For example, Yang et al. (2001) showed that only a small proportion of bacteria associated with plant surfaces cultivate on standard media. Second, our volatile bioassays were conducted on bacterial strains, while in nature bacteria rarely occur in isolation. Although our technique allowed for direct comparisons of bacteria responses to volatiles at different concentrations, competition in natural communities would likely lead to exclusion of strains over time (Lindow and Brandl 2003). Here, *P. agglomerans* is of particular interest because it was present across all tissues and is known to outcompete and suppress other tissue dwelling bacteria (Johnson et al. 2000). Suggestive of this competition was that more strains from female-phase flowers were identified as *P. agglomerans* compared to male-phase flowers in our protandrous species however, more detailed sampling and controlled experiments are necessary to determine if bacteria competition occurs in the flowers as they age. Third, we tested racemic linalool here while *P. digitalis* produces only (*S*)-(+)-linalool in the flowers. Some basic testing shows anti-microbial properties of linalool enantiomers may vary (Schmidt et al. 2005) so our results should be viewed with caution. Fourth, the ‘nectar’ was constant throughout our trials unlike the depleted and replenished nectar resource of flowers, which could have affected the growth and density patterns we observed. However, our results do show a marked difference between the two main nectar volatiles with racemic linalool having a much greater effect on bacteria than methyl nicotinate, even at such elevated levels of methyl nicotinate. These results are suggestive of a role in plant–microbe interactions for linalool in our system.

## Conclusion

Here we show that linalool could defend *P. digitalis* tissues by slowing the growth rate of specific bacteria, raising the possibility that plant–microbe interactions may influence selection on this volatile. Our work adds to the growing body of research that suggests alternative functions beyond pollinator attraction for floral volatiles. Further work could help to address why flowers produce floral scents when scent is expected to play little role in plant–pollinator interactions, such as bird-pollinated or self-pollinated flowers.

## Electronic supplementary material

Below is the link to the electronic supplementary material.

Supplementary material 1 (XLSX 46 KB)

Supplementary material 2 (XLSX 224 KB)
